# Differential Toll-Like Receptor-Signalling of *Burkholderia pseudomallei* Lipopolysaccharide in Murine and Human Models

**DOI:** 10.1371/journal.pone.0145397

**Published:** 2015-12-21

**Authors:** Tassili A. F. Weehuizen, Joann L. Prior, Thomas W. van der Vaart, Sarah A. Ngugi, Sergey A. Nepogodiev, Robert A. Field, Liesbeth M. Kager, Cornelis van ‘t Veer, Alex F. de Vos, W. Joost Wiersinga

**Affiliations:** 1 Center for Infection and Immunity Amsterdam (CINIMA), Academic Medical Center, Amsterdam, the Netherlands; 2 Center for Experimental and Molecular Medicine (CEMM), Academic Medical Center, Amsterdam, the Netherlands; 3 Defence Science and Technology Laboratory, Porton Down, Salisbury, United Kingdom; 4 John Innes Centre, Norwich Research Park, Colney, United Kingdom; 5 Department of Internal Medicine, Division of Infectious Diseases, Academic Medical Center, Amsterdam, the Netherlands; University of Toledo School of Medicine, UNITED STATES

## Abstract

The Gram-negative bacterium *Burkholderia pseudomallei* causes melioidosis and is a CDC category B bioterrorism agent. Toll-like receptor (TLR)-2 impairs host defense during pulmonary *B*.*pseudomallei* infection while TLR4 only has limited impact. We investigated the role of TLRs in *B*.*pseudomallei*-lipopolysaccharide (LPS) induced inflammation. Purified *B*.*pseudomallei*-LPS activated only TLR2-transfected-HEK-cells during short stimulation but both HEK-TLR2 and HEK-TLR4-cells after 24 h. In human blood, an additive effect of TLR2 on TLR4-mediated signalling induced by *B*.*pseudomallei*-LPS was observed. In contrast, murine peritoneal macrophages recognized *B*.*pseudomallei*-LPS solely through TLR4. Intranasal inoculation of *B*.*pseudomallei*-LPS showed that both TLR4-knockout(^-/-^) and TLR2x4^-/-^, but not TLR2^-/-^ mice, displayed diminished cytokine responses and neutrophil influx compared to wild-type controls. These data suggest that *B*.*pseudomallei*-LPS signalling occurs solely through murine TLR4, while in human models TLR2 plays an additional role, highlighting important differences between specificity of human and murine models that may have important consequences for *B*.*pseudomallei*-LPS sensing by TLRs and subsequent susceptibility to melioidosis.

## Introduction

The category B bioterrorism agent, as classified by the Center for Disease Control and Prevention, *Burkholderia pseudomallei* is a facultative intracellular Gram-negative bacterium and the causative agent of melioidosis [1–3]. Melioidosis, an important cause of sepsis in Southeast Asia and Northern Australia, is characterized by pneumonia and the formation of multiple abscesses and is associated with case fatality rates of up to 40% despite appropriate antibiotic treatment [1, 2]. Among the multiple putative virulence factors that have been described for *B*.*pseudomallei*, such as *Burkholderia* lethal factor 1, type III and VI secretion systems, capsular polysaccharide and flagella, lipopolysaccharide (LPS) stands out for its omnipresence and the high antibody titers which are generated against it in patients [4, 5]. Yet, in contrast to other Gram-negative pathogens, the LPS of *B*.*pseudomallei* is considered only weakly inflammatory [6]. In general LPS, which consists of lipid A, the core-oligosaccharide and the outer O-polysaccharide, plays an important role in cell integrity and in signalling to the host innate immune response [7, 8].

There are several lines of evidence that suggest an important role for LPS in the pathogenesis of melioidosis. First, high levels of antibodies to LPS are associated with a better outcome in patients with melioidosis suggesting that LPS needs to be recognized for an appropriate immune response [4, 5]. In addition, the *B*.*pseudomallei* mutant strain SRM117 lacking an O-antigen is less virulent in animal models utilising hamsters, guinea pigs and diabetic rats when compared to the parent strain. This might be caused by the reduced resistance to opsonization, rendering the bacterium more susceptible to killing by macrophages and neutrophils [9–12]. Furthermore, administration of monoclonal antibodies (mAb) specifically directed against LPS of *B*.*pseudomallei* proved to be protective in a murine model of inhalational melioidosis [13, 14]. However, the LPS of *B*.*pseudomallei* is reported to be less immunostimulatory in comparison to LPS derived from pathogenic *Enterobacteriaceae* [6]. In addition, systemic LPS levels at admission do not correlate with outcome in patients with melioidosis [15, 16].

In general the structure of the lipid A moiety of LPS is well conserved between strains and its presence sensed by the Toll-like receptor (TLR)-4 complex upon which the immune response is initiated [8]. While adequate cellular recognition of LPS can aid in the clearance of the invading pathogen, overstimulation of host cells by LPS can lead to septic shock. However, not all Gram-negative bacteria produce LPS that can be recognized by the TLR4/MD2 complex, possibly as a result of their non-hexa-acyl lipid A structure [8, 17]. For instance, *Porphyromonas gingivalis*-LPS contains multiple lipid A species that functionally interact with both TLR2 and TLR4 and *Leptospiral* LPS is predominantly recognized by TLR2 [18, 19].

Conflicting evidence exists regarding whether the LPS of *B*.*pseudomallei* signals through TLR2 or TLR4. We previously reported that a LPS compound derived from *B*.*pseudomallei* strain 1026b extracted by the hot aqueous-phenol method [20] was recognized by TLR2 and not TLR4 in Human Embryonic Kidney (HEK293) cells stably transfected with CD14, CD14-TLR2, or CD14-TLR4/MD-2 [21]. In contrast, purified LPS derived from strain K96243 was shown to signal through TLR4 using the same in vitro model [22]. However, the *in vivo* role of TLR recognition of *B*.*pseudomallei-* LPS has not yet been investigated.

In the present study we aimed to investigate the importance of LPS as a virulence factor of *B*.*pseudomallei* and the contribution of TLR2 and TLR4 in *B*.*pseudomallei-* LPS induced inflammation. We found that LPS of *B*.*pseudomallei* induces a strong inflammatory response. Moreover, we established that TLR4 is the main receptor for LPS of *B*.*pseudomallei* in murine *in vitro* and *in vivo* models. Remarkably, in human *in vitro* models TLR2 plays an additional role in *B*.*pseudomallei-* LPS-signalling.

## Materials and Methods

### Isolation and purification of LPS

LPS was extracted from *B*.*pseudomallei* 1026b and purity was confirmed using a combination of previously published methods [23, 24]. Cell pellets of to log-phase grown *B*. *pseudomallei* 1026b were digested for 16 hours at 4°C with 15,000 Units of lysozyme (Sigma-Aldrich, Dorset, UK) per mg of bacteria, prior to digestion with 20 μg/ml of DNase I and RNase A (Sigma-Aldrich) for a further 16 h at room temperature. This was followed by a Proteinase K (Sigma-Aldrich) (50mg/ml) digestion step for 6 hours at room temperature. The LPS was then treated by a modified hot phenol method. Briefly, the cell paste and 90% phenol (Sigma-Aldrich) were independently heated to 70°C before adding the phenol to the cell paste at a 1:1 ratio. The mixture was vigorously stirred by hand whilst maintaining 70°C. This mixture was dialysed against water until no phenol remained, after which it was lyophilised. A further round of Proteinase K, DNase I and RNase A digestions preluded a final ultracentrifugation step at 100,000 x g for 3 hours. Pellets were solubilised with distilled water before being dialysed into further distilled water. The final product was purified by SDS-PAGE on a 12.5% separating gel and visualised by silver staining.

Protein contamination in the LPS preparation was tested by silver staining, Coomassie blue staining (Bio-Rad, Hercules, CA) and BCA assay (Thermo Fisher Scientific, Rockford, IL). In order to further confirm the purity of the LPS, different concentrations of LPS-binding cationic antimicrobial polymyxin B (PMB) [25, 26] were used in a stimulation assay using the murine alveolar macrophage cell line MH-S (American Type Culture Collection, Rockville, MD).

### Isolation and mass spectrometric analysis of lipid A

Lipid A components of *B*.*pseudomallei*-LPS of strains 1026b and K96243 [27] were obtained by treatment of LPS as described [20]. Matrix-assisted laser desorption/ionisation time-of-flight mass spectrometry (MALDI-TOF-MS) of the lipid A component of *B*.*pseudomallei*-LPS of strains 1026b and K96243 was performed in negative-ion mode on a Bruker UltraFlex MALDI-TOF/TOF instrument using 2,4,6-trihydroxyacetophenon as a matrix. Sample preparation and analysis were conducted as described [28].

### Cells and cell culture conditions

HEK-293 cells stably expressing human CD14-TLR2 or CD14-TLR4/MD2 (kindly provided by Dr. Douglas Golenbock, University of Massachusetts Medical School, Worcester, Massachusetts) were used as described previously [21]. HEK-293 cells were cultured in DMEM medium enriched with 10% FCS, 1% L-glutamine, 1% PenStrep (100 units penicillin, 100 ug streptomycin; Life technologies, Bleiswijk, the Netherlands) and blasticidine (10 ug/ml: InvivoGen San Diego, CA). HEK-cells were seeded at 2.5 x 10^5^ /ml and stimulated with LPS of *B*.*pseudomallei* 1026b (100 ng/ml), ultrapure LPS of *Escherichia coli* O111:B4 (100 ng/ml: InvivoGen), PAM3CSK4 (100 ng/ml, InvivoGen) or medium for 6 or 24 hours. Supernatants were harvested and stored at -20°C until assayed for interleukin (IL)-8.

In additional experiments, lipoprotein lipase **(**LPL) (Sigma-Aldrich) was added to HEK-cells during LPS stimulation. The murine alveolar macrophage cell line MH-S was grown in RPMI 1640 medium supplemented with 10% FCS, 1% L-glutamine, 1% PenStrep and 0.05 mM 2-mercaptoethanol (Sigma-Aldrich) and seeded at 1.5 x10^6^/ml, followed by stimulation with the stimuli as described above in the presence or absence of LPS-binding polymyxin B (25–100 ug/ml; Sigma-Aldrich). Supernatants were harvested at 6 and 24 h and stored at -20°C until assayed for murine tumor necrosis factor (TNF)-α. For selected experiments whole blood derived from healthy volunteers was collected; for these studies inhibitory anti-human TLR2 (2500 ng/ml) and TLR4 (1000 ng/ml) antibodies were used (InvivoGen) as described [29]. Dose-response curves were performed prior to electing the appropriate dose of each stimulus and for the different cells used (data not shown).

### Mice and murine ex vivo stimulation experiments

TLR2 deficient (TLR2^-/-^) and TLR4^-/-^ mice were kindly provided by Dr Shizuo Akira (Osaka University, Japan) and were backcrossed at least six times to a C57BL/6 background. TLR2^-/-^/TLR4^-/-^ double knock-out (TLR2x4^-/-^) mice were generated by intercrossing TLR2^-/-^ and TLR4^-/-^ mice [30]. Pathogen-free wild-type (WT) C57BL/6 mice were purchased from Charles River (Maastricht, the Netherlands). Age (10–12 week) and sex-matched animals were used in all experiments.

Blood and peritoneal macrophages were harvested from WT, TLR2^-/-^ and TLR4^-/-^ mice. Blood was then transferred to V-bottom 96-well plates (Greiner Bio-One, Frickenhausen, Germany; 100 ul/well) and directly stimulated with 10^7^ CFU/ml of heat-killed *B*. *pseudomallei* 1026b or its O-antigen lacking mutant SRM117 [9, 11], LPS of *B*. *pseudomallei* 1026b (100 ng/ml), LPS of *E*. *coli* (100 ng/ml), PAM3CSK4 (100 ng/ml) or RPMI 1640 for 6 h or 24 h. Peritoneal macrophages were harvested by 5 ml peritoneal lavage with sterile PBS. Cells were resuspended in RPMI 1640 enriched with 1% L-glutamine, 1% penicillin/streptomycin, and 10% FCS and seeded in a 96-well plate at a concentration of 0.5 x 10^6^ /ml and. The next day the cells were washed with RPMI 1640 to remove non-adherent cells and incubated with the same stimuli as used for whole blood; a MOI of 50 of heat-killed *B*. *pseudomallei* 1026b and SRM117 was used. Dose-response curves for these stimuli were performed prior to these experiments (data not shown).

### LPS-induced lung inflammation

Lung inflammation was induced in mice as described previously [31]. In short, mice were lightly anesthetized by inhalation of isoflurane, after which 50 μl of LPS of *B*.*pseudomallei* 1026b (200 ug/ml) dissolved in PBS was administered intranasally. This dose of 10 μg LPS intranasally was based on a previous dose-finding experiment (data not shown). After 6 h, mice were sacrificed by bleeding from the vena cava inferior. Bronchoalveolar lavage with two 0.5 ml aliquots of sterile saline was performed as described [31]. The 6 h time point was chosen as the ideal time to simultaneously assess cyto- and chemokine alterations and leukocyte recruitment [31]. BAL fluid (BALF) differential cell counts were performed on Giemsa-stained cytospin preparations. After centrifuging the BALF samples at 650x *g* for 10 minutes, supernatants were harvested and stored at -20°C for cytokine analysis.

### Assays

Human IL-8 levels were measured in the supernatants of HEK-cell supernatant by ELISA (Biosource, Etten-Leur, the Netherlands). Release of TNF–α, interleukin-6 (IL-6) and cytokine-induced neutrophil chemo-attractant (KC) was measured in supernatant and BALF by ELISA (R&D Systems, Minneapolis, MN) in accordance with the manufacturer’s recommendations. Cell counts in BALF were determined using a Beckman Coulter Counter (Miami, United States).

### Statistical analysis

Data were analyzed using GraphPad Prism for Windows (version 5.01; GraphPad Software). Data were analyzed by Mann-Whitney U test. When > 2 groups were treated with the same stimuli, a Kruskall-Wallis test was performed first. Data are expressed as mean ± SEM values. P-values < 0.05 were considered statistically significant.

### Ethics and safety statement

The Animal Care and Use of Committee of the University of Amsterdam approved all experiments, which were conducted according to national guidelines (DIX 102324/25). Mice were sacrificed under ketamine (Eurovet, Bladel, the Netherlands) and medetomine (Pfizer, Capelle, Netherlands) or isoflurane anesthesia, and all efforts were made to minimize suffering. In addition, the biosafety committee of the University of Amsterdam approved all *in vitro* experiments.

## Results

### Extraction and purity of *B*.*pseudomallei-*LPS

**Fig 1:**
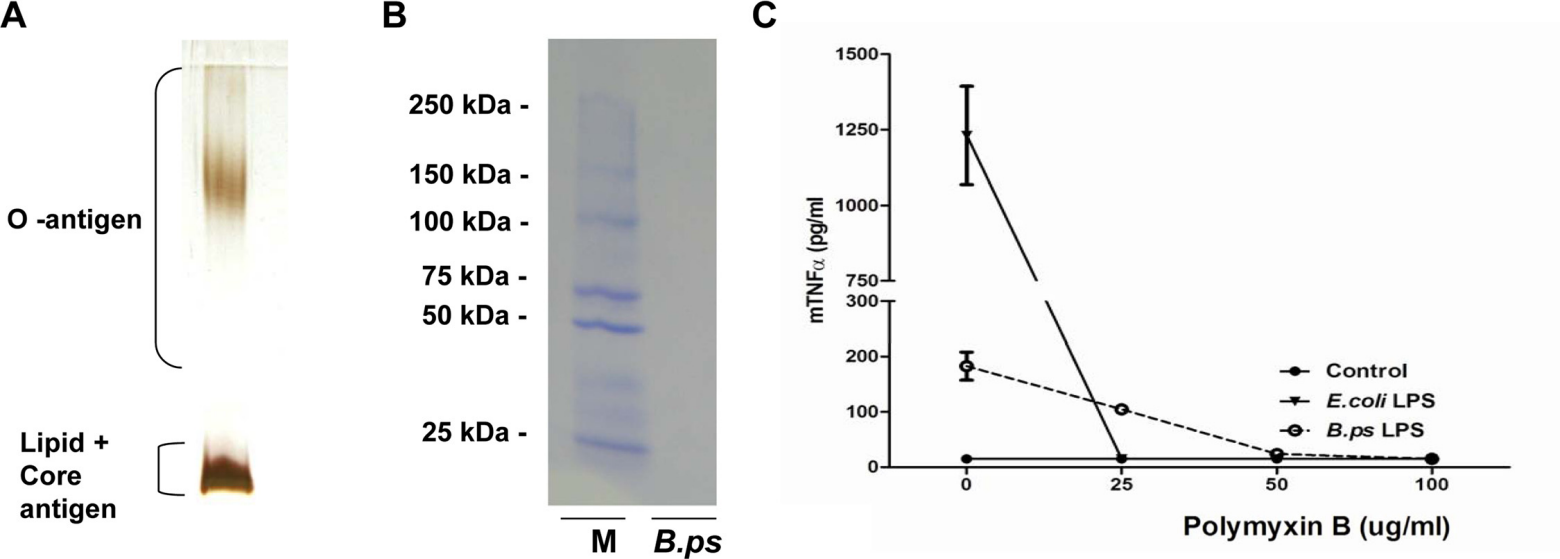
Successful extraction and purification of *B*.*pseudomallei-*LPS. LPS from *B*.*pseudomallei* 1026b was purified by a modified hot phenol-water extraction method including proteinase K treatment. 10 μl of LPS (0.5 mg/ml) was fractionated by SDS-PAGE electrophoresis followed by silver staining (A) which showed the characteristic ladder pattern of LPS banding of Gram-negative bacteria, without any indications of protein contamination (detection limit of 0.5–5 ng) and Coomassie blue staining (B) which demonstrated no protein contamination as well (detection limit 50 ng). Contamination of the extracted LPS was also assessed by the addition of LPS-binding Polymyxin B (PMB) (C). For this purpose, the murine alveolar macrophage cell line MH-S was stimulated with RPMI 1640 medium [56], LPS of *E*. *coli* 0111:B4 or *B*.*pseudomallei* 1026b and incubated with increasing concentrations of PMB for 6h, followed by TNF-α measurement in the supernatant. Results are representative for three independent experiments. (M = ladder, B.ps = *B*.*pseudomallei*-LPS)

To ensure that the LPS of *B*.*pseudomallei* 1026b was not degraded after extraction, 5 μg of LPS (0.5 mg/ml) was run on a 12.5% gel and silver-stained, which demonstrated a typical LPS pattern [32, 33] (Fig 1A) and no protein contamination. In addition, the LPS was assessed for protein contamination by an enhanced BCA assay and Coomassie Blue staining (Fig 1B) which were negative (data not shown). Even though *B*.*pseudomallei* itself is not or just slightly growth-inhibited by its presence [34], PMB was able to completely inhibit the inflammatory response induced by 50 μg/ml of purified *B*.*pseudomallei-* LPS in the murine alveolar cell line MH-S (Fig 1C). Of note, PMB by itself did not have an effect on MH-S cell viability, neither did it have any effect of the inflammatory response caused by PAM3CSK4, a synthetic TLR2-ligand (data not shown). These results underscore that there are no potential immune stimulatory contaminating agents present in the LPS preparation used.

Since it is known that the structure of the lipid A component of LPS is of importance recognition by TLRs [35], the isolated lipid A components of *B*.*pseudomallei* strains 1026b and K96243 were structurally analyzed by MALDI TOF mass spectrometry. As expected, the analyses revealed a high degree of heterogeneity of these isolates, which gave spectra that were very similar (S1 Fig). We confirmed that the lipid A species in the LPS of both *B*. *pseudomallei* K96234 and *B*. *pseudomallei* 1026b were predominantly tetra-acylated with a small proportion that were penta-acylated (Table 1, S1 and S2 Figs), which is in line with previous studies [16]

**Table 1:** The main MALDI-TOF-MS negative-ion peaks of lipid A component of *B*. *pseudomallei* 1026b and their proposed interpretation.

Observed ion (*m/z*)	Acyl Substitution	Proposed fatty acid, phosphate and Ara_4_N composition
1218.2	triacyl	2 × C16:0(3-OH), C14:0, 2 × P
1364.5	tetraacyl	C14:0(3-OH), 2 × C16:0(3-OH), C14:0, 1 × P
1445.5	tetraacyl	C14:0(3-OH), 2 × C16:0(3-OH), C14:0, 2 × P
1460.4	tetraacyl	2 × C14:0(3-OH), 2 × C16:0(3-OH), 2 × P
1575.5	tetraacyl	C14:0(3-OH), 2 × C16:0(3-OH), C14:0, 2 × P, Ara4N
1671.8	pentaacyl	2 × C14:0(3-OH), 2 × C16:0(3-OH), C14:0, 2 × P
1686.5	pentaacyl	3 × C14:0(3-OH), 2 × C16:0(3-OH), 2 × P

Lipid A of *B*. *pseudomallei* 1026b was analyzed by negative-ion MALDI-TOF-MS. Several negative-ion peaks were found and interpreted. Several peaks corresponding to single-charged lipid A ions were found and interpreted by comparison with calculated *m/z* values of variously substituted species of lipid A shown in S2 Fig. Complex pattern of molecular ion peaks indicative of a heterogeneous mixture of species is often observed in MS spectra of lipid A isolated from bacterial LPS, for example *B*.*pseudomallei*, *B*.*thailandensis* [16] and *B*. *Mallei* [20]. *(m/z)* = mass-to-charge ratio.

### *B*.*pseudomallei-*LPS functionally interacts with both human TLR2 and -4

**Fig 2:**
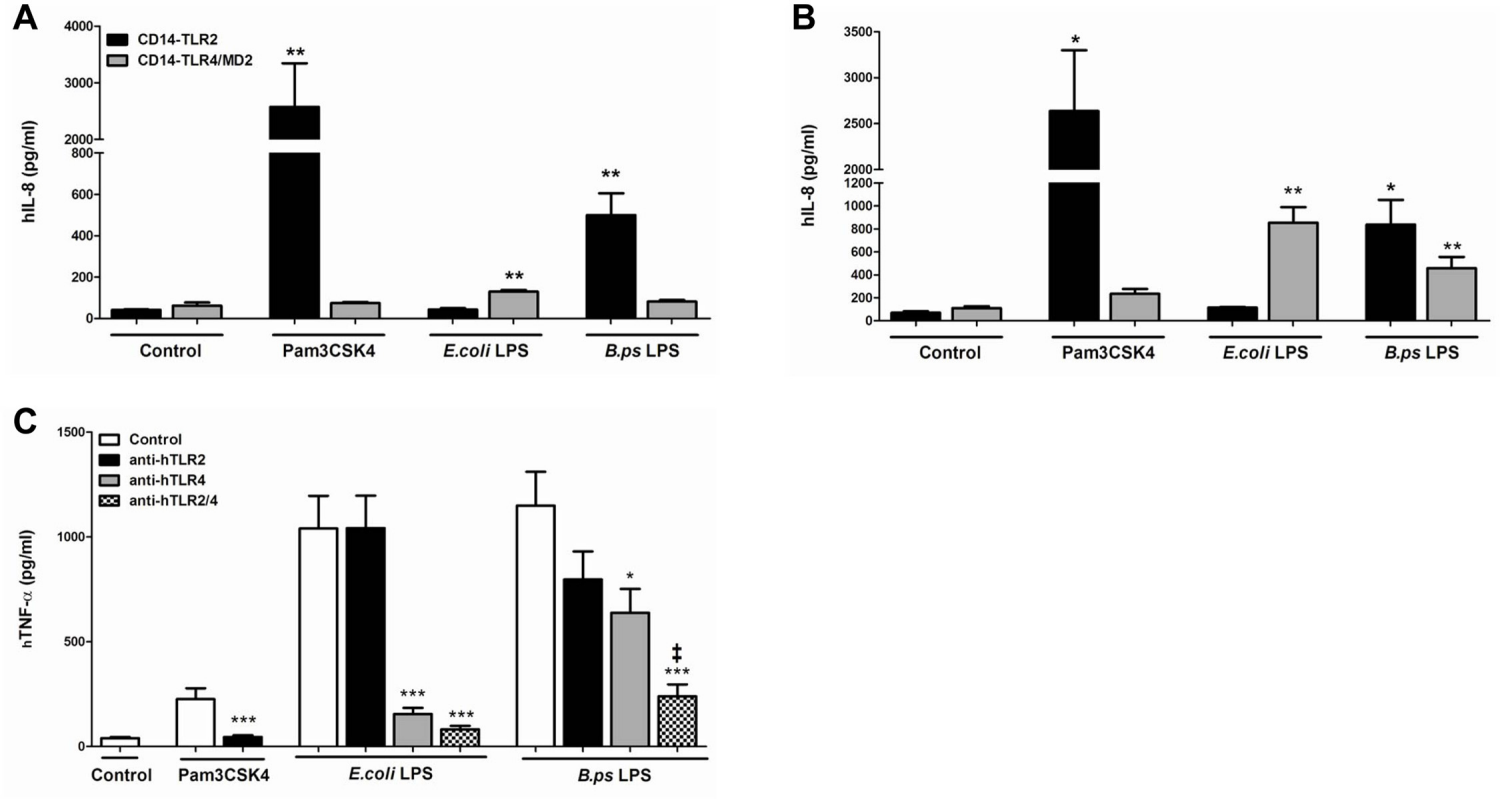
LPS of *B*.*pseudomallei* signals via both TLR2 and TLR4 in *in vitro* human models. Human Embryonic Kidney (HEK)-293 cells, stably transfected with either CD14-TLR2 or CD14-TLR4/MD2 were stimulated with purified LPS of *B*.*pseudomallei* 1026b (100 ng/ml), LPS of *E*. *coli* 0111:B4 (100 ng/ml), PAM3CSK4 (100 ng/ml) or DMEM+ 10% FCS for 6h (A) or 24h (B) before measurement of interleukin (IL)-8 in the supernatant (n = 3). Human whole blood was pre-treated for 30 minutes with respectively RPMI 1640 medium, anti-TLR2 antibody (2500 ng/ml), anti-TLR4 antibody (1000 ng/ml) or both antibodies and hereafter stimulated with purified LPS of *B*.*pseudomallei 1026b* (100 ng/ml), LPS of *E*. *coli* 0111:B4 (100 ng/ml), PAM3CSK4 (100 ng/ml) or RPMI 1640 for 6h after which TNF was measured (C) (n = 3). An additive effect of TLR2 on TLR4 mediated signalling induced by *B*.*pseudomallei*-LPS was observed. Data are presented as means ± SEM. Results of two or three independent experiments were pooled. Mann-Whitney- U tests were performed. *P< 0.05; **P< 0.01, ***P< 0.001 vs. control (or no antibodies). ^*‡*^ *P*< 0.05 anti-TLR2 and 4 vs. anti-TLR4 alone.

In order to investigate via which TLR the LPS of *B*.*pseudomallei* signals, we first stimulated HEK-293 cells stably transfected with either human CD14-TLR2 or CD14-TLR4/MD2. In line with our previous experiments we confirmed that after 6 h of stimulation *B*.*pseudomallei-*LPS signals via TLR2 and not TLR4 in this cell system [21] (Fig 2A). However, after 24 hours of stimulation the LPS of *B*.*pseudomallei* clearly interacted with both TLR2 and TLR4 (Fig 2B). To examine the signalling more carefully, we next performed the reverse experiment using human whole blood derived from healthy volunteers in combination with inhibitory anti-human TLR2 or TLR4 antibodies. After pre-incubation with an anti-TLR2 or anti-TLR4 antibody, we stimulated whole blood with *B*.*pseudomallei-*LPS (100 ng/ml) for 6 h. In concordance with the experiments with HEK-cells, these experiments demonstrated that *B*.*pseudomallei-*LPS signals mainly via TLR4 in a human *in vitro* system (Fig 2C). Interestingly, anti-TLR2 antibody tended to reduce the inflammatory response, but this was not significant (p = 0.09). However, combining the anti-TLR2/4 antibodies did demonstrate an additional effect of TLR2 blockade. With regard to the inflammatory response induced by the whole bacterium, we did not observe any inhibitory effect on the inflammatory response induced by *B*.*pseudomallei* when these antibodies were used (data not shown). In all experiments, *E*.*coli-*LPS, used as positive control, was confirmed to signal only through TLR4. In addition, these results showed that the extent of cytokine responses induced by *B*.*pseudomallei-*LPS was comparable to those induced by *E*.*coli-*LPS.

### TLR4 –but not TLR2—deficiency attenuates the murine cellular inflammatory response to *B*.*pseudomallei-*LPS

**Fig 3:**
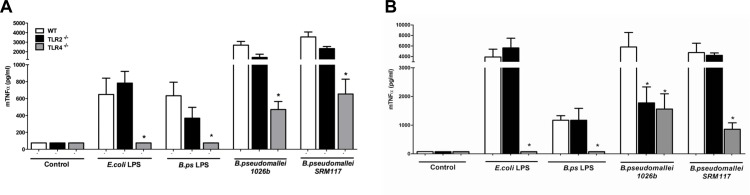
LPS of *B*.*pseudomallei* signals solely via murine TLR4. Murine whole blood (A) and peritoneal macrophages (B) harvested from wild-type (WT), TLR2^-/-^ and TLR4^-/-^ mice were stimulated for 24h with RPMI 1640 + 10% FCS medium, LPS of *E*. *coli* 0111:B4 (100 ng/ml), LPS of *B*.*pseudomallei* (100 ng/ml), heat-killed *B*.*pseudomallei* 1026b (100 ng/ml), or its O-antigen lacking mutant SRM117 (both 10^7^ CFU/ml) before measurement of murine TNF-α (n = 4). Following a Kruskal-Wallis test, Mann-Whitney U-tests were performed. Data are means ± SEM. Results are representative of two or three independent experiments. *P< 0.05 vs. WT.

After demonstrating the important role of both TLR2 and TLR4 in the signalling of *B*.*pseudomallei-*LPS in human cell models, we next examined the TLR-signalling in an *ex vivo* murine model. Whole blood and primary peritoneal macrophages harvested from WT, TLR2^-/-^ and TLR4^-/-^ mice were collected and stimulated with *B*.*pseudomallei*-LPS, heat-killed *B*.*pseudomallei* and its mutant SRM117 that lacks the O-antigen for 6 and 24 h. In this model, *B*.*pseudomallei-*LPS behaved in the same way as *E*. *coli-*LPS: TLR4^-/-^ but not TLR2^-/-^ murine whole blood (Fig 3A) and peritoneal macrophages (Fig 3B) showed a completely abolished TNF-α response upon stimulation. Interestingly, reduced TNF-α release was observed in whole blood derived from TLR4^-/-^ but not TLR2^-/-^ mice in response to both heat-killed *B*.*pseudomallei* and the SRM117 mutant (Fig 3A). Surprisingly, stimulation of TLR2^-/-^ peritoneal macrophages with heat-killed *B*.*pseudomallei* demonstrated an equally hampered response as TLR4 deficient macrophages (Fig 3B). An even more remarkable finding was that this TLR-2-mediated response was O-antigen-dependent since it differed from the response to stimulation with mutant strain SRM117. Despite being less virulent in animal models [9], *B*. *pseudomallei* mutant SRM117 induced a similar inflammatory response in WT blood and macrophages.

In a separate set of experiments whole blood and peritoneal macrophages derived from TLR2/4 double knock-out mice were exposed to *B*.*pseudomallei-* LPS; however no additional effect of TLR2 or double TLR deficiency was observed in the inflammatory response (data not shown). Taken together, this set of experiments showed that–in sharp contrast to the human studies—*B*.*pseudomallei-*LPS interacts with TLR4 and not TLR2 in *ex vivo* murine models.

### TLR4 acts as the main TLR receptor for neutrophil recruitment during acute murine lung inflammation induced by *B*.*pseudomallei*-LPS

**Fig 4:**
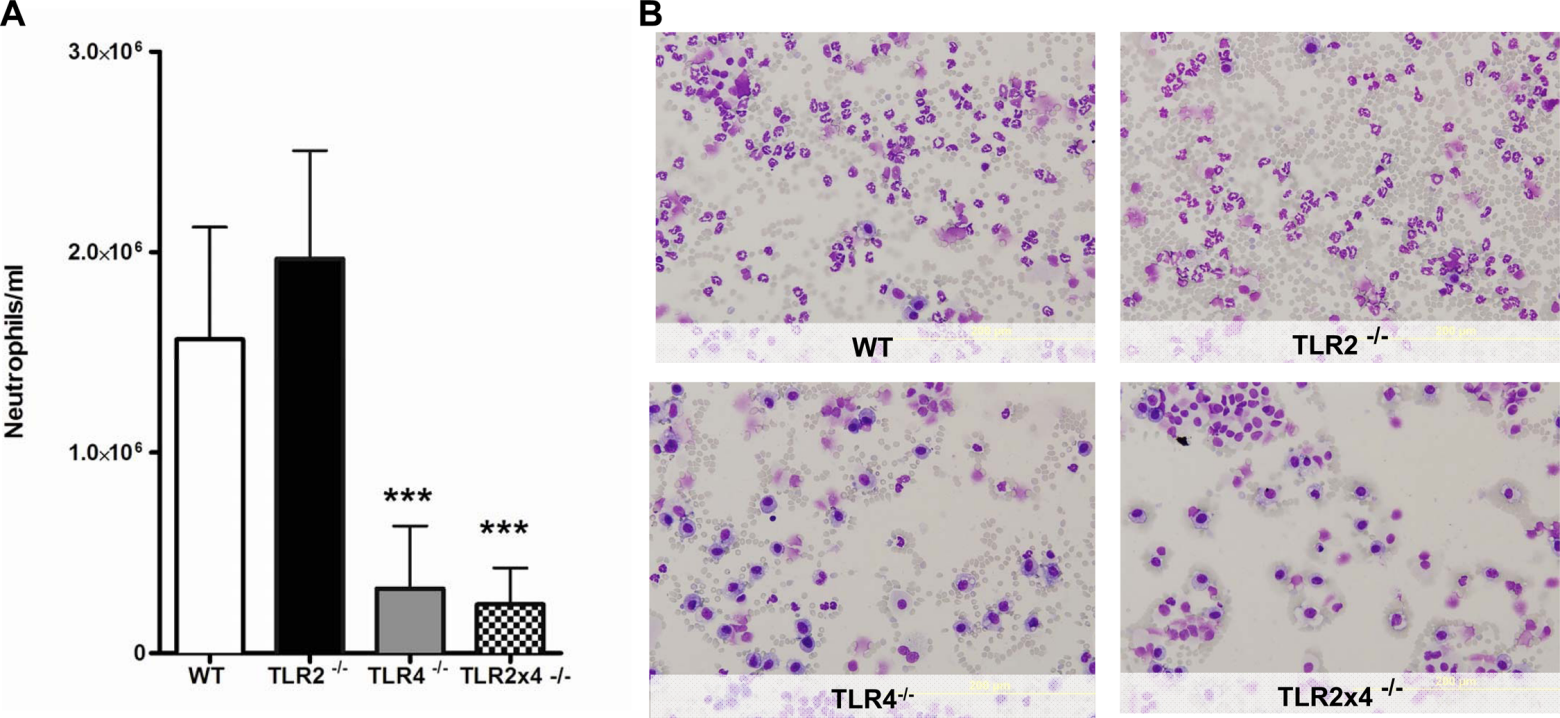
*B*.*pseudomallei-* LPS-induced neutrophil influx is dependent on TLR4 in mice. Wild-type **(**WT), TLR2^-/-^, TLR4^-/-^ and TLR2x4^-/-^ mice were inoculated intranasally with 10 ug LPS of *B*.*pseudomallei* 1026b and analysed 6h later for lung neutrophil influx in bronchoalveolar fluid (BALF) (A). Representative Giemsa stained BALF slides are shown of WT, TLR2^-/-^, TLR4^-/-^ and TLR2x4^-/-^ mice obtained 6h post-LPS administration (magnification 20x) (B). Following a Kruskal-Wallis test, Mann-Whitney U-tests were performed. Data are presented as means ± SEM. Results of two independent experiments were pooled (n = 16 per group). ***P< 0.001 vs. WT.

We have previously shown that intranasal inoculation with *B*.*pseudomallei* causes significant and rapid inflammation and neutrophil, but not monocyte, recruitment toward the lung [36]. To evaluate the role of *B*.*pseudomallei-*LPS during pulmonary inflammation, we intranasally inoculated WT, TLR2^-/-^, TLR4^-/-^ and TLR2x4^-/-^ mice with *B*.*pseudomallei-*LPS to examine polymorphonuclear leukocyte (PMN) influx. BALF was obtained 6 h after LPS administration since this time point is representative for both neutrophil influx and local cytokine/chemokine release. No differences in pulmonary neutrophil influx were seen between WT and TLR2^-/-^ mice (Fig 4). However, when compared to WT mice, *B*.*pseudomallei* LPS-induced PMN influx was equally and strongly reduced in TLR4^-/-^ and TLR2x4^-/-^ mice (Fig 4).

### *B*.*pseudomallei-*LPS induced pulmonary inflammation in mice is TLR4 dependent

**Fig 5:**
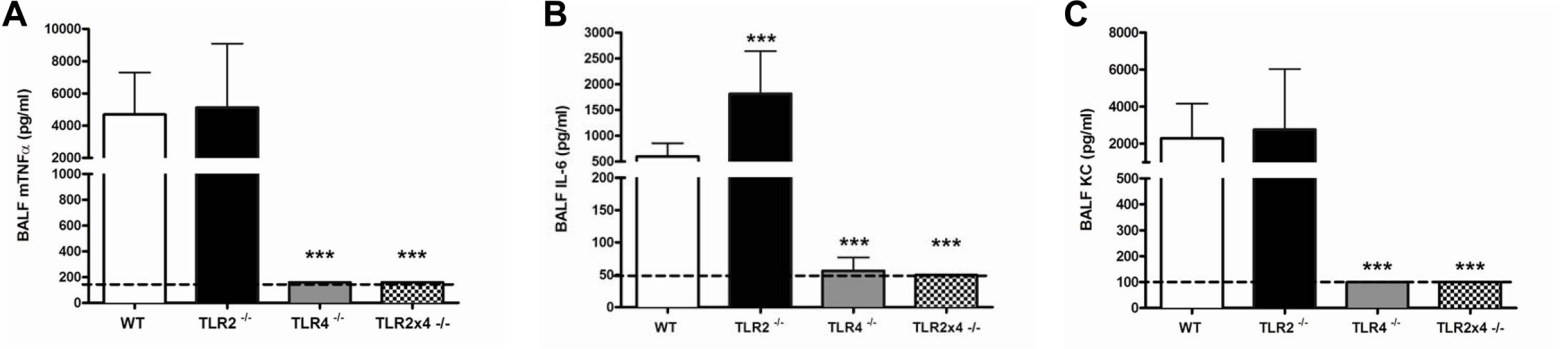
TLR4^-/-^ and TLR2x4^-/-^, but not TLR2^-/-^ mice, display diminished cytokine responses to *B*.*pseudomallei* LPS-induced pulmonary inflammation in mice. mTNF-α (A), interleukin (IL)-6 (B) and KC (C) levels were determined in bronchoalveolair fluid (BALF) of wild-type (WT), TLR2^-/-^, TLR4^-/-^ and TLR2x4^-/-^ mice 6h post-intranasal administration of 10 ug LPS of *B*.*pseudomallei*. Following a Kruskal-Wallis test, Mann-Whitney U-tests were performed. Data are presented as means ± SEM. N = 7 or 9 per group and experiments were performed in duplicate. *** *P*< 0.001 vs. WT.

Intranasal administration of *B*.*pseudomallei-*LPS resulted in a steep increase in the concentrations of TNF-α and IL-6 in BALF (Fig 5A and 5B). TLR4, but not TLR2, did profoundly influence this characteristic inflammatory response. *B*.*pseudomallei-*LPS induced TNF-α and IL-6 levels determined 6 h after administration were found to be significantly lower in TLR4^-/-^ and TLR2x4^-/-^ mice, but not in TLR2^-/-^ mice when compared to WT mice (Fig 5A and 5B). Of note, TLR2 deficiency resulted in increased IL-6 levels when compared to WT; however no additional effect of TLR2 was seen in the TLR2x4^-/-^ mice when compared to the TLR4^-/-^ mice. In correlation with the strongly diminished neutrophil influx in TLR4^-/-^ and TLR2x4^-/-^ mice, an equally sharp decline in the production of the neutrophil attracting chemokine KC in BALF was seen (Fig 5C). These findings further confirm our conclusion that TLR4 acts as the main *in vivo* receptor for *B*.*pseudomallei*-LPS in mice.

## Discussion

LPS recognition by the host is of crucial importance for the initiation of a swift host immune response to Gram-negative bacteria [37]. In the case of melioidosis, a main cause of community-acquired Gram-negative sepsis in Southeast Asia and Northern Australia however, essential insights on both the role of LPS as a putative virulence factor and its recognition by the innate immune system remain ill defined [1, 2]. We now studied the recognition and contribution of TLR2 and TLR4 in *B*.*pseudomallei*-LPS induced inflammation, linking *in vitro* experiments using human and murine cells with mouse studies involving LPS-induced pulmonary inflammation. Using these models we found that the LPS of *B*.*pseudomallei* induces a strong inflammatory response that is comparable to the response elicited by *E*.*coli-*LPS, supporting previous data [38]. Moreover, we established that TLR4 is the main receptor for LPS of *B*.*pseudomallei* in murine *in vitro* and *in vivo* models. Remarkably, in human *in vitro* models TLR2 has an additional role in the recognition of *B*.*pseudomallei-*LPS. Taken together these results further characterize *B*.*pseudomallei-*LPS as a key driver of the innate immune response in melioidosis. In addition, these findings underscore important species differences in the specificity of LPS-TLR interactions, which means caution should be applied when generalizations are made when extrapolating from murine disease models to humans [39].

TLRs and CD14 are key pattern-recognition receptors of the innate immune system that are capable of recognizing ‘pathogen-associated molecular patterns’ (PAMPs) and form a crucial link between innate and adaptive immunity. The key role of TLRs in the pathogenesis of melioidosis is underscored by the finding that mice deficient in myeloid-differentiation-primary-response-gene-88 (MyD88), which is the key signalling adaptor protein for all TLRs, except for TLR3, show a strongly accelerated lethality upon intranasal infection with *B*.*pseudomallei* [40]. TLR4 has been implicated as the canonical LPS-receptor for Gram-negative bacteria, recognizing the lipid A part of LPS [41]. Efficient LPS signalling requires both the LPS-binding protein (LBP) and the surface-anchored receptor CD14, which has demonstrated to be involved in the recognition of *B*.*pseudomallei* [42]—and the extracellular protein MD2. Furthermore, the glycosylation status of LPS influences its signalling; smooth LPS, which contains the complete O-antigen, requires CD14 for its detection, whereas the O-antigen lacking rough LPS and lipid A do not [43]. *B*.*pseudomallei-*LPS seems to be largely conserved across this species. LPS profiling of >700 *B*.*pseudomallei* isolates using SDS-PAGE showed that most isolates had a ‘typical’ ladder pattern of extracted LPS while a minority had an ‘atypical’ or rough pattern [44]. *B*.*pseudomallei-*LPS diversity may correlate with differential immunopathogenicity and virulence, such as biofilm formation among *B*.*pseudomallei* strains [32, 45]. Observed differences in TLR-signalling of LPS might further be explained by different three-dimensional shapes of the lipid A part [46], which are due to a different disaccharide backbone, the presence or absence of carboxyl or phosphate groups, numbers and (non)symmetrical position of acyl-groups [47]. Conical shaped lipid A such as that of *E*.*coli* signals via TLR4, while cylindrical shaped lipid A has been described to signal via TLR2 [46]. *B*.*pseudomallei*’s lipid A is known to be penta-acylated, while the lipid A part of most other virulent Gram-negative bacteria often possesses 6 acyl groups, and contains a fatty acyl mutation (C_14:0_(2-OH) [16]. In our study we found similar acylation patterns for the lipid A part of both *B*. *pseudomallei* 1026b and K96243, making it less likely that TLR signalling of LPS differs between these *B*.*pseudomallei* strains. A role for TLR2 in the recognition of *B*.*pseudomallei-*LPS would not be unique: it is known that the LPS of *Legionella pneumophila*, *Leptospira interrogans* and *Porphyromonas gingivalis* can signal via TLR2 [8, 18, 19]. Remarkably, the TLR2-mediated response was O-antigen-dependent since it differed from the response to stimulation with the O antigen mutant strain SRM117. One could hypothesize that differences in the recognition of smooth LPS (which includes O-antigen) and rough LPS (which lacks O-antigen) may play a role herein. Another explanation might be that O-antigen deficiency leads to changes in the expression of other *B*. *pseudomallei* associated PAMPs and thus TLR ligands.

We now demonstrate that, depending on the model used, *B*.*pseudomallei-*LPS recognition occurs solely via TLR4 (murine models) or via a combination of TLR2 and -4 (human models. Our results underscore previous studies that have demonstrated important differences in TLR-signalling in humans and mice [48, 49]. Moreover, in a study on *Pseudomonas aeruginosa* infection and cystic fibrosis (CF) it has been shown that *P*. *aeruginosa* adapts itself to the CF airway by synthesizing both hexa- and penta-acylated LPS. Human TLR4 can discriminate between these LPS-forms, whereas murine TLR4 cannot [50]. This is thought to be mediated by an 82-aa region of human TLR4 that varies highly across species [50]. Using mass spectrometry, we confirmed that lipid A of *B*.*pseudomallei* has a tetra- and penta-acylated saccharide backbone, which could be the reason the TLR4 response to *B*.*pseudomallei*-LPS is much stronger in mice than in humans. In this respect it should be mentioned that TLR4-region genetic variants in humans are associated with susceptibility to melioidosis [51]. Of note however, this effect could also be attributed to a non-LPS TLR4 effect, since this promiscuous receptor not only recognizes LPS but also recognizes numerous endogenous danger signals called ‘danger-associated molecular patterns’ (DAMPs) [52].

Pneumonia is the most common manifestation of acute melioidosis [1, 53]. Our study is, to our knowledge, the first to provide insights into the ability of *B*.*pseudomallei-* LPS to elicit broncho-alveolar inflammation. We demonstrated that *B*.*pseudomallei-* LPS elicits a profound inflammatory response, comparable to that induced by instillation of an equal dose of *E*.*coli-*LPS [31]. *B*.*pseudomallei-*LPS induced the release of TNF-α, IL-6 and KC into BALF together with a marked influx of neutrophils into the alveolar space. Together with our *in vitro* data that show that the extent of cytokine responses induced by *B*.*pseudomallei-*LPS was comparable to those induced by *E*.*coli*-LPS, these results challenge current views that *B*.*pseudomallei-*LPS is only weakly inflammatory, which is in line with recent reports [38]. This effect was dependent on TLR4. TLR2 deficiency did not play a role in murine LPS recognition: either TLR2 deficiency on its own or TLR2/TLR4 double deficiency did not significantly influence these inflammatory parameters.

Our study has several limitations. Potential contamination with TLR2-stimulating agents such as lipopeptides is a well-known problem during LPS purification [54]. The LPS of *B*.*pseudomallei* used in this study was obtained by a modified aequous hot-phenol re-extraction method and we have confirmed its integrity and purity by both silver and Coomassie staining and a BCA protein assay. To further test potential contamination, in supplementary experiments, we studied whether digestion of *B*.*pseudomallei* LPS with lipoprotein lipase **(**LPL) could abolish the response in HEK-TLR2 cells; however LPL treatment did not impact on TLR-signalling by *B*.*pseudomallei*-LPS (S3 Fig). No TLR2 dependent response was observed in the murine *in vitro* stimulation experiments. In addition, the LPS-binding antimicrobial PMB was able to completely inhibit the inflammatory response induced by the purified *B*.*pseudomallei-*LPS. It has been demonstrated that TLR signalling might differ in established cell lines and primary cells [55]; this can be of particular relevance for our observation of *B*.*pseudomallei-* LPS—TLR2 signalling when using a TLR overexpressing system of HEK-cells. However, when we performed the reverse experiment on human whole blood with the use of blocking TLR antibodies we still observed a significant effect of TLR2 on the signalling of *B*.*pseudomallei-*LPS. Lastly, it should be noted that TLR expression is known to vary considerably across mammalian species [49]. Murine and human inflammatory responses towards LPS stimulation can be markedly different [39]. Our study now further accentuates the implications of these findings.

In summary, we here demonstrate that LPS of *B*.*pseudomallei* is capable of inducing a strong inflammatory response in both human and murine models underscoring an important role for LPS as a virulence factor of *B*.*pseudomallei*. We conclude that the main receptor of *B*.*pseudomallei*-LPS is TLR4. However, in human *ex vivo* models there is an additional role of TLR2 in the signalling of *B*.*pseudomallei-*LPS. These data emphasize species differences in TLR-signalling in men and mice and clearly imply that extrapolation of murine data to humans should be carried out with great caution. Future research on these species discrepancies is therefore needed.

## Supporting Information

S1 Fig Negative-ion mode MALDI-TOF mass spectra of lipid A components of *B*. *pseudomallei* 1026b (*A*) and K96243 (*B*). Based on peaks in these spectra and comparison to literature data [16] acylation patterns of lipid A were proposed (Table 1). The spectra of the observed negative-ion peaks were similar for both strains. *(m/z)* = mass-to-charge ratio. *Da* = dalton.
(TIF)

S2 Fig Calculated *m/z* values for proposed structures of substituted lipid A backbone. (TIFF)

S3 Fig Lipoprotein lipase treatment does not significantly alter TLR-2 signalling of *B*.*pseudomallei*-LPS. Human Embryonic Kidney (HEK)-293 cells, stably transfected with either CD14-TLR2 or CD14-TLR4/MD2 were stimulated with purified LPS of *B*.*pseudomallei* 1026b (100 ng/ml), LPS of *E*. *coli* 0111:B4 (100 ng/ml), PAM3CSK4 (100 ng/ml) or DMEM+ 10% FCS. Subsequently, lipoprotein lipase (200 or 2000 ng/ml) was added to the culture. 24h post-stimulation supernatant was collected and interleukin (IL)-8 was measured by ELISA (n = 4). Data are presented as means ± SEM and were analysed by Kruskall- Wallis analysis followed by Mann-Whitney- U tests. *P< 0.05 compared to control.
(DOCX)
