# Supplementary figures and images for: Differential Toll-Like Receptor-Signalling of Burkholderia pseudomallei Lipopolysaccharide in Murine and Human Models
Source: PLoS One. 2015 Dec 21;10(12):e0145397. doi: 10.1371/journal.pone.0145397 (PMC4687033; doi:10.1371/journal.pone.0145397)

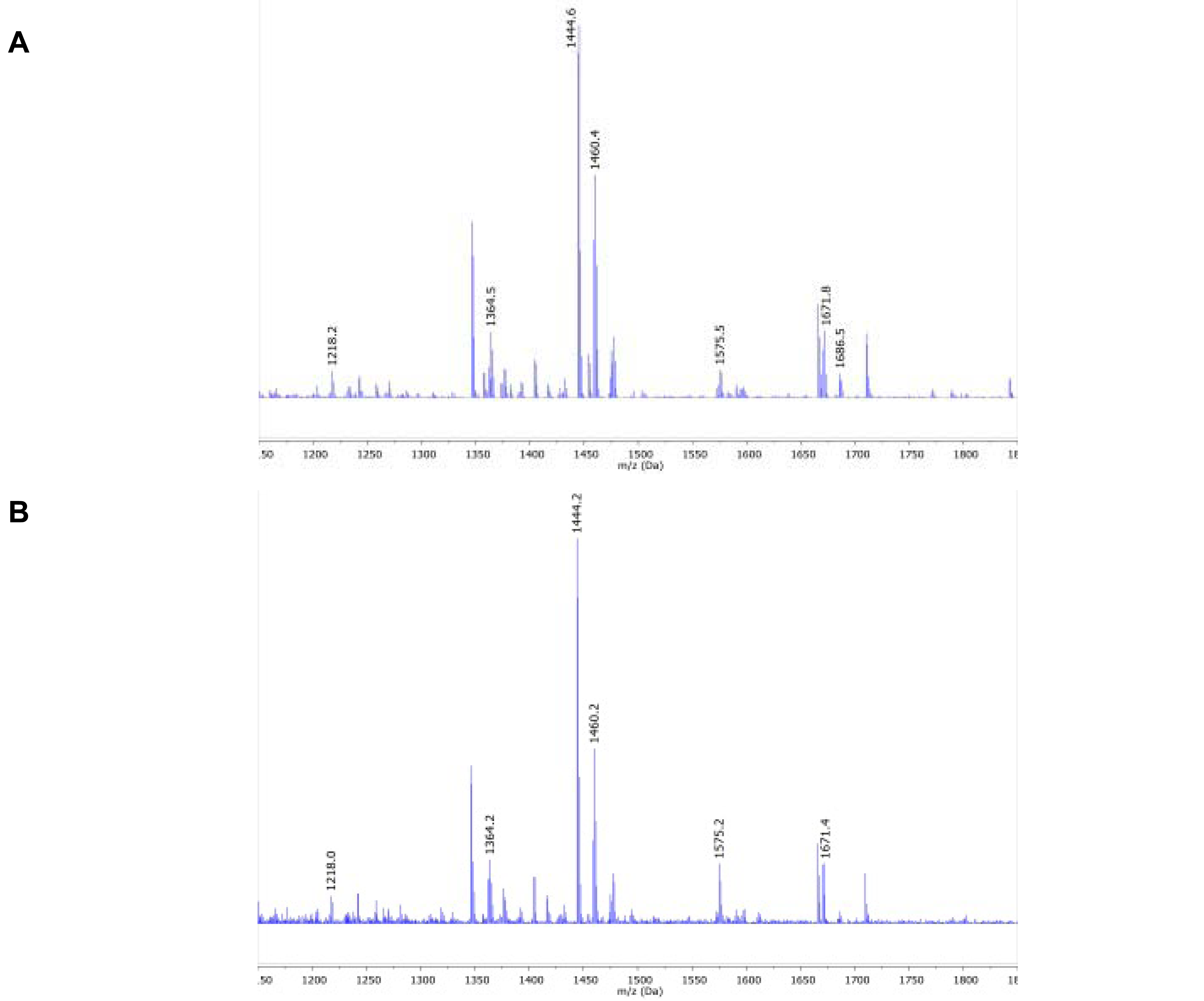

Supplement: S1 Fig — Based on peaks in these spectra and comparison to literature data [16] acylation patterns of lipid A were proposed (Table 1). The spectra of the observed negative-ion peaks were similar for both strains. (m/z) = mass-to-charge ratio. Da = dalton. (TIF) [file pone.0145397.s001.tif]

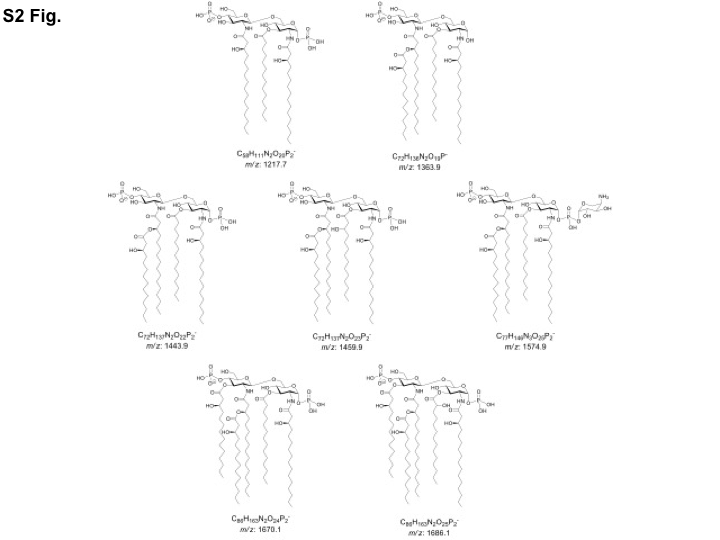

Supplement: S2 Fig — (TIFF) [file pone.0145397.s002.tiff]
