# Supplementary material for: Differential Toll-Like Receptor-Signalling of Burkholderia pseudomallei Lipopolysaccharide in Murine and Human Models
Source: PLoS One. 2015 Dec 21;10(12):e0145397. doi: 10.1371/journal.pone.0145397 (PMC4687033; doi:10.1371/journal.pone.0145397)
Supplement: S3 Fig — Human Embryonic Kidney (HEK)-293 cells, stably transfected with either CD14-TLR2 or CD14-TLR4/MD2 were stimulated with purified LPS of B.pseudomallei 1026b (100 ng/ml), LPS of E. coli 0111:B4 (100 ng/ml), PAM3CSK4 (100 ng/ml) or DMEM+ 10% FCS. Subsequently, lipoprotein lipase (200 or 2000 ng/ml) was added to the culture. 24h post-stimulation supernatant was collected and interleukin (IL)-8 was measured by ELISA (n = 4). Data are presented as means ± SEM and were analysed by Kruskall- Wallis analysis followed by Mann-Whitney- U tests. *P< 0.05 compared to control. (DOCX) [file pone.0145397.s003.docx]

**Figure S3: Lipoprotein lipase treatment does not significantly alter TLR-2 signalling of *B.pseudomallei*-LPS.**


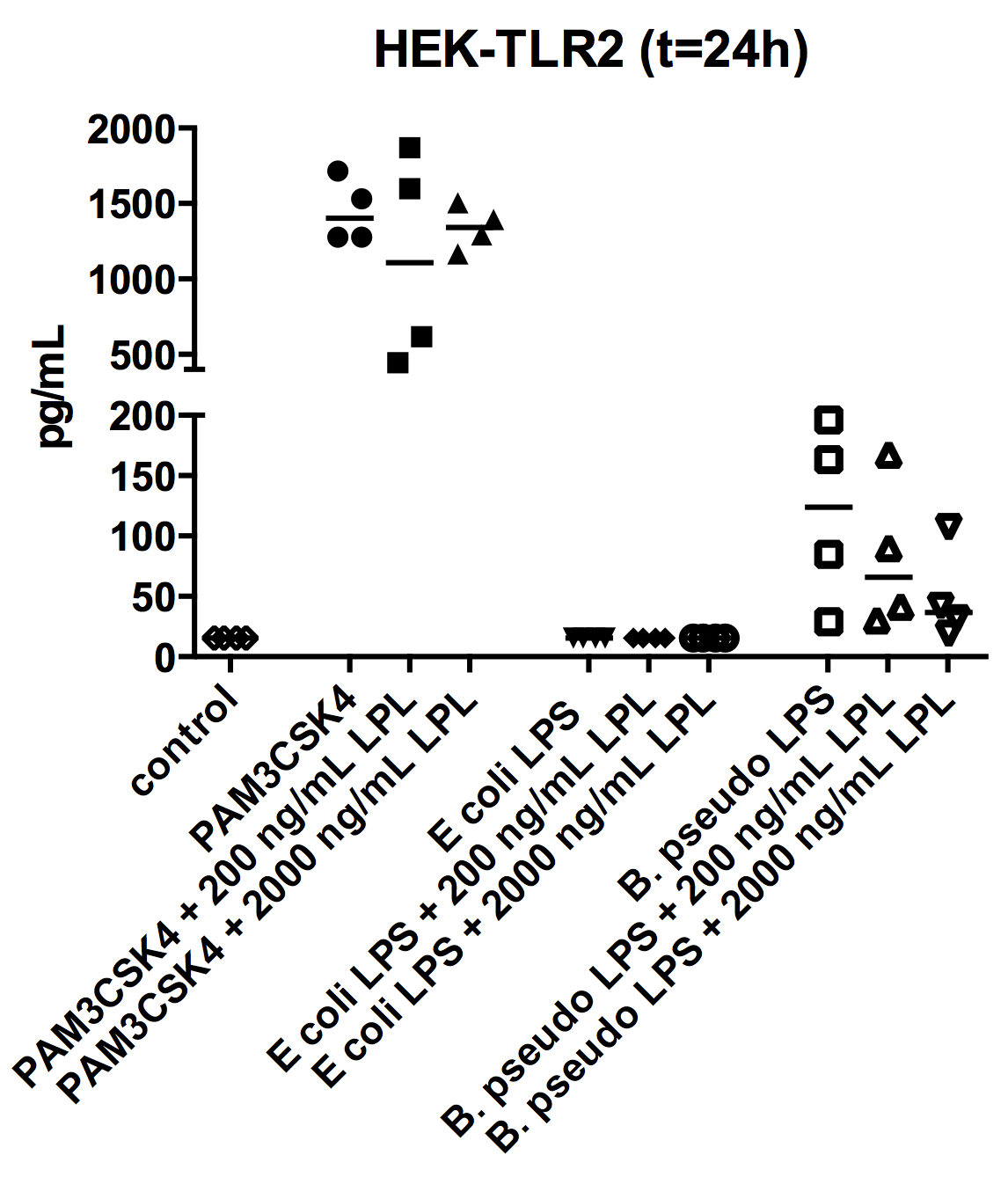
**A**


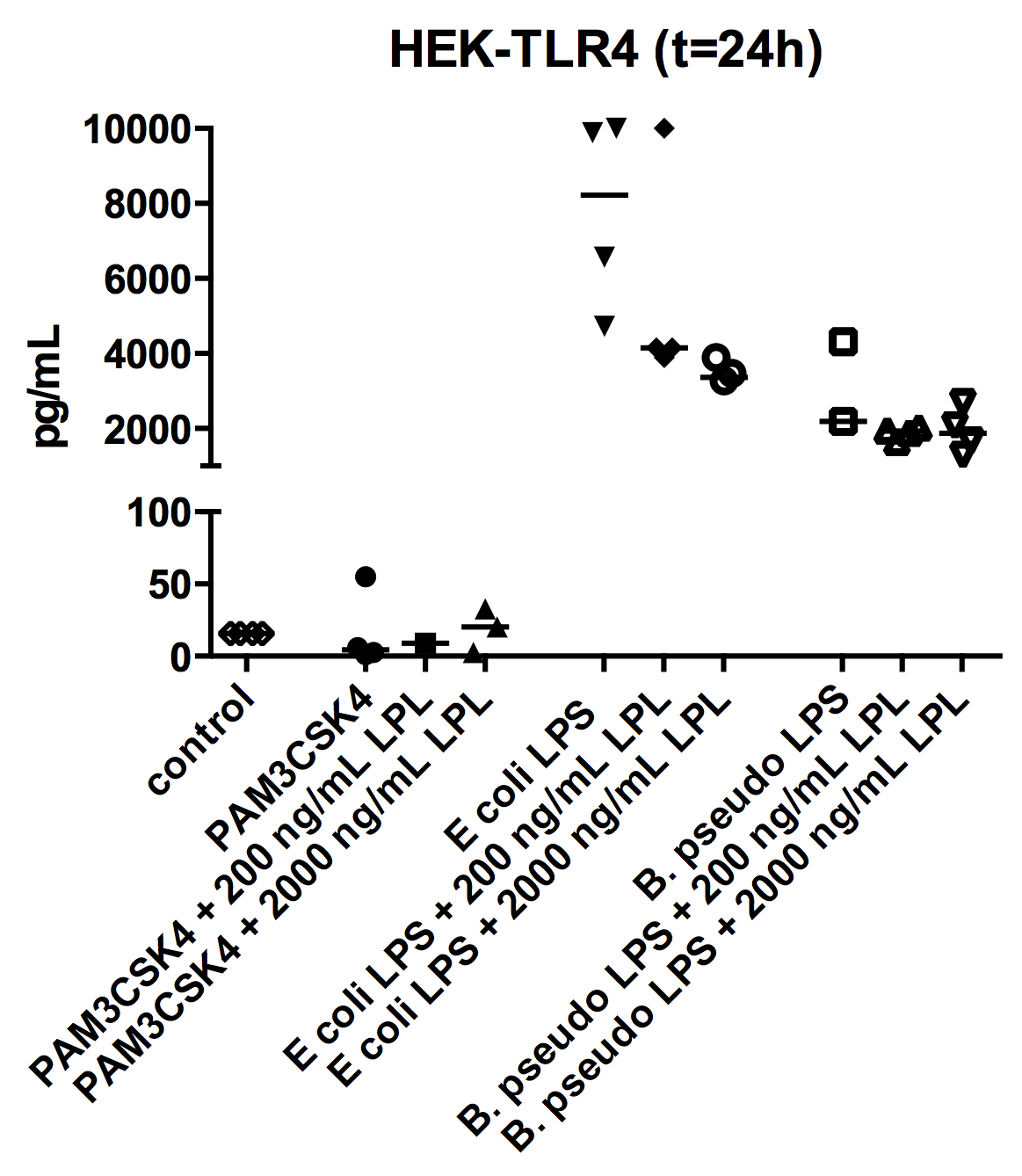
**B**

Human Embryonic Kidney (HEK)-293 cells, stably transfected with either CD14-TLR2 or CD14-TLR4/MD2 were stimulated with purified LPS of *B.pseudomallei* 1026b (100 ng/ml), LPS of *E. coli* 0111:B4 (100 ng/ml), PAM3CSK4 (100 ng/ml) or DMEM+ 10% FCS. Subsequently, lipoprotein lipase (200 or 2000 ng/ml) was added to the culture.

24h post-stimulation supernatant was collected and interleukin (IL)-8 was measured by ELISA (n=4). Data are presented as means ± SEM and were analysed by Kruskall- Wallis analysis followed by Mann-Whitney- U tests. **P*< 0.05 compared to control.
